# A phase II trial of regorafenib in patients with metastatic and/or a unresectable gastrointestinal stromal tumor harboring secondary mutations of exon 17

**DOI:** 10.18632/oncotarget.17310

**Published:** 2017-04-21

**Authors:** Chun-Nan Yeh, Ming-Huang Chen, Yen-Yang Chen, Ching-Yao Yang, Chueh-Chuan Yen, Chin-Yuan Tzen, Li-Tzong Chen, Jen-Shi Chen

**Affiliations:** ^1^ Department of Surgery, GIST Team, Chang Gung Memorial Hospital and University, Taoyuan 333, Taiwan; ^2^ School of Medicine, National Yang-Ming University, Taipei 112, Taiwan; ^3^ Department of Oncology, Taipei Veterans General Hospital, Taipei 112, Taiwan; ^4^ Department of Internal Medicine, Division of Hematology-Oncology, Chang Gung Memorial Hospital, Kaohsiung Medical Center, Kaohsiung 833, Taiwan; ^5^ Department of Surgery, National Taiwan University Hospital, National Taiwan University College of Medicine, Taipei 100, Taiwan; ^6^ Department of Pathology and Laboratory Medicine, Cathay General Hospital, Taipei 100, Taiwan; ^7^ National Institute of Cancer Research, National Health Research Institutes, Tainan 70456, Taiwan; ^8^ Division of Hematology-Oncology, GIST Team, Linkou Chang Gung Memorial Hospital and Chang Gung University, Taoyuan 333, Taiwan

**Keywords:** regorafenib, GIST, exon 17

## Abstract

**Background:**

Gastrointestinal stromal tumors (GISTs) are caused by the constitutive activation of KIT or platelet-derived growth factor receptor alpha (PDGFRA) mutations. Imatinib selectively inhibits KIT and PDGFR, leading to disease control for 80%–90% of patients with metastatic GIST. Imatinib resistance can occur within a median of 2–3 years due to secondary mutations in KIT. According to preclinical studies, both imatinib and sunitinib are ineffective against exon 17 mutations. However, the treatment efficacy of regorafenib for patients with GIST with exon 17 mutations is still unknown.

**Patients and Methods:**

Documented patients with GIST with exon 17 mutations were enrolled in this study. Patients received 160 mg of oral regorafenib daily on days 1–21 of a 28-day cycle. The primary end point of this trial was the clinical benefit rate (CBR; i.e., complete or partial response [PR], as well as stable disease [SD]) at 16 weeks. The secondary end points of this study included progression free survival (PFS), overall survival, and safety.

**Results:**

Between June 2014 to May 2016, 18 patients were enrolled (15 of which were eligible for response evaluation). The CBR at 16 weeks was 93.3% (14 of 15; 6 PR and 8 SD). The median PFS was 22.1 months. The most common grade 3 toxicities were hand-and-foot skin reactions (10 of 18; 55.6%), followed by hypertension (5 of 18; 27.8%).

**Conclusion:**

Regorafenib significantly prolonged PFS in patients with advanced GIST harboring secondary mutations of exon 17. A phase III trial of regorafenib versus placebo is warranted.

**Trial registration:**

This trial is registered atClinicalTrials.gov in November 2015, number NCT02606097.

**Key message:**

This phase II trial was conducted to assess the efficacy and safety of regorafenib in patients with GIST with exon 17 mutations. The results provide strong evidence that regorafenib significantly prolonged PFS in patients with advanced GIST harboring secondary mutations of exon 17.

## INTRODUCTION

Gastrointestinal stromal tumors (GISTs) are the most common mesenchymal tumors of the gastrointestinal tract [1]. In the West and in Taiwan, GISTs are commonly associated with mutations in the KIT receptor tyrosine kinase, leading to constitutive activation [2–3]. Imatinib mesylate (IM) is an oral agent that specifically inhibits the BCR-ABL gene, as well as the KIT and PDGFR tyrosine kinases [4]. IM was previously reported to induce a partial response (PR) or stable disease (SD) in more than 80% of patients with unresectable or metastatic GIST, whereas primary resistance to IM occurred in approximately 15% of patients with GIST [5–6]. While most patients with advanced GIST benefit from IM, many patients subsequently develop IM resistance, with the median time of 24–36 months after IM treatment, leading to further disease progression [7–8]. The mechanisms of acquired IM resistance in GIST are not well understood. A variety of possible causes for this resistance have been suggested, including KIT second mutations, KIT genomic amplification, an alternative receptor tyrosine kinase activation in the absence of KIT expression, decreased IM bioavailability after chronic IM therapy, the cessation of IM when the disease is stable and measurable, and subtherapeutic IM levels [9–12].

KIT exon 17 mutations contributed to 30%–40% of KIT secondary mutations responsible for GIST patient resistance to imatinib or sunitinib [13–14]. Although a new tyrosine kinase inhibitor has been developed, the best management for patients with GIST who develop KIT exon17 mutations after IM treatment remains unclear. Since exon 17 is the activation loop (A-loop) encoding region of the KIT kinase, its mutation makes it clinically resistant to all of the new tyrosine kinase inhibitors, including sunitinib, sorafenib, dasatinib, and nilotinib. Recently, one phase II trial demonstrated the treatment efficacy of regorafenib in four patients with IM-and sunitinib-resistant KIT activation loop mutations D820Y and N822K. All four patients achieved a clinical benefit, with two patients having a positive response to regorafenib during the study (with the progression free survival [PFS] being 11 and 7 months, respectively). One patient experienced disease progression at 5.7 months [12].

Based on the above results and the unmet medical needs of patients with GIST harboring exon 17 mutations, we conducted a phase II trial to assess the efficacy and safety of regorafenib in this population of patients.

## RESULTS

### Patient characteristics

Between June 2014 and May 2016, 18 patients who met the inclusion criteria were enrolled in this study. The demographic and pathologic characteristics of the patients are described in Table 1. The median age was 59 years (range: 36–71 years). Fourteen patients (77.8%) were men, and 12 patients (66.7%) had an Eastern Cooperative Oncology Group (ECOG) performance status (PS) of 1. Fourteen patients (77.8%) harbored both exon 11 and exon 17 mutations, including missense mutation at codon 816 (1 of 14), 820 (4 of 14), 822 (6 of 14), and 823 (3 of 14). Two patients (11.1%) harbored both exon 9 and exon 17 mutations (missense mutation at codon 822; 2 of 2) and two patients (11.1%) harbored exon 11, 13, and 17 mutations (missense mutation at codon 823; 2 of 2). All the patients had received IM treatment for more than 18 months and 10 patients (55.6%) had also received sunitinib. Twelve patients (66.7%) at enrollment had disease progression following prior targeted therapy, while six patients (33.3%) had SD.

**Table 1:** Demographic data and treatment outcomes of regorafenib treatment for advanced GIST patients harboring exon 17 mutations (*N = 18*)

Age; median (range)	59 (36-71)
Gender	
Male: Female	14:4
ECOG	
0	6
1	12
Mutation status	
Exons 11 and 17	14
Exons 9 and 17	2
Exons 11 and 13 and 17	2
Previous tyrosine kinase inhibitor	
Imatinib	8
Imatinib then sunitinib	10
Duration of previous imatinib therapy	
≦6 months	0
6–18 months	0
≧18 months	18
Duration of previous sunitinib therapy	
≦6 months	1
6–18 months	3
≧18 months	6
Best response to regorafenib at 16 weeks	
PR	6
SD	8
PD	1
NA	3
CBR (PR + SD)	14/15 (93.3%)
Median progression-free survival (months)	22.1

PR: partial response; SD: stable disease; PD: progressive disease; NA: not available; CBR: clinical benefit rate.

### Patient characteristics of historical cohort

For comparison to the patients in this study, we collected data on 15 patients with GIST who had a confirmed exon 17 mutation but did receive regorafenib treatment as a historical cohort. The demographic and pathologic characteristics of these patients are described in Supplementary Table 1. The median age was 59 years (range: 35–72 years). Ten patients (66.7%) were men. Ten patients (66.7%) harbored both exon 11 and exon 17 mutations, two patients (13.3%) harbored both exon 9 and exon 17 mutations, and three patients (20%) harbored exon 11, 13, and 17 mutations.

### Efficacy

**Figure 1:**
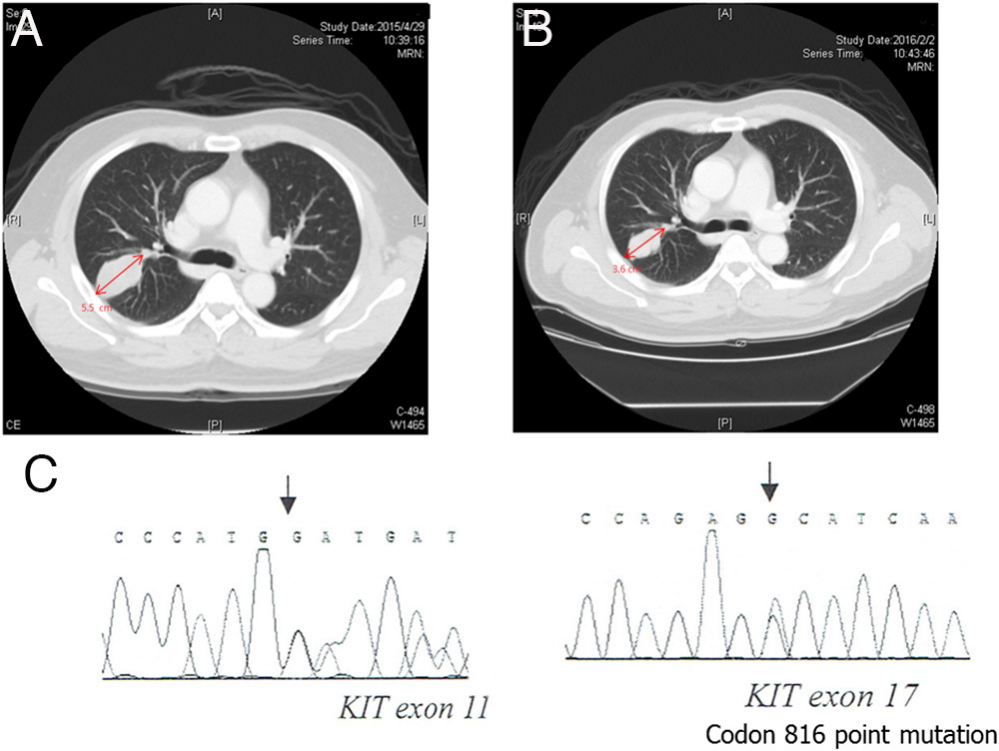
Regorafenib showed a treatment response in a patient with gastrointestinal stromal tumor with lung metastasis harboring a secondary mutation of exon 17 **(A)** Pretreatment CT with contrast showed a mass measuring 5.5cm over the right lung. **(B)** Post-treatment CT with contrast showed the mass had decreased to 3.6 cm over the right lung. **(C)** Direct sequence analysis of DNA from this specimen revealed a missense mutation at D816E in exon 17.

**Figure 2:**
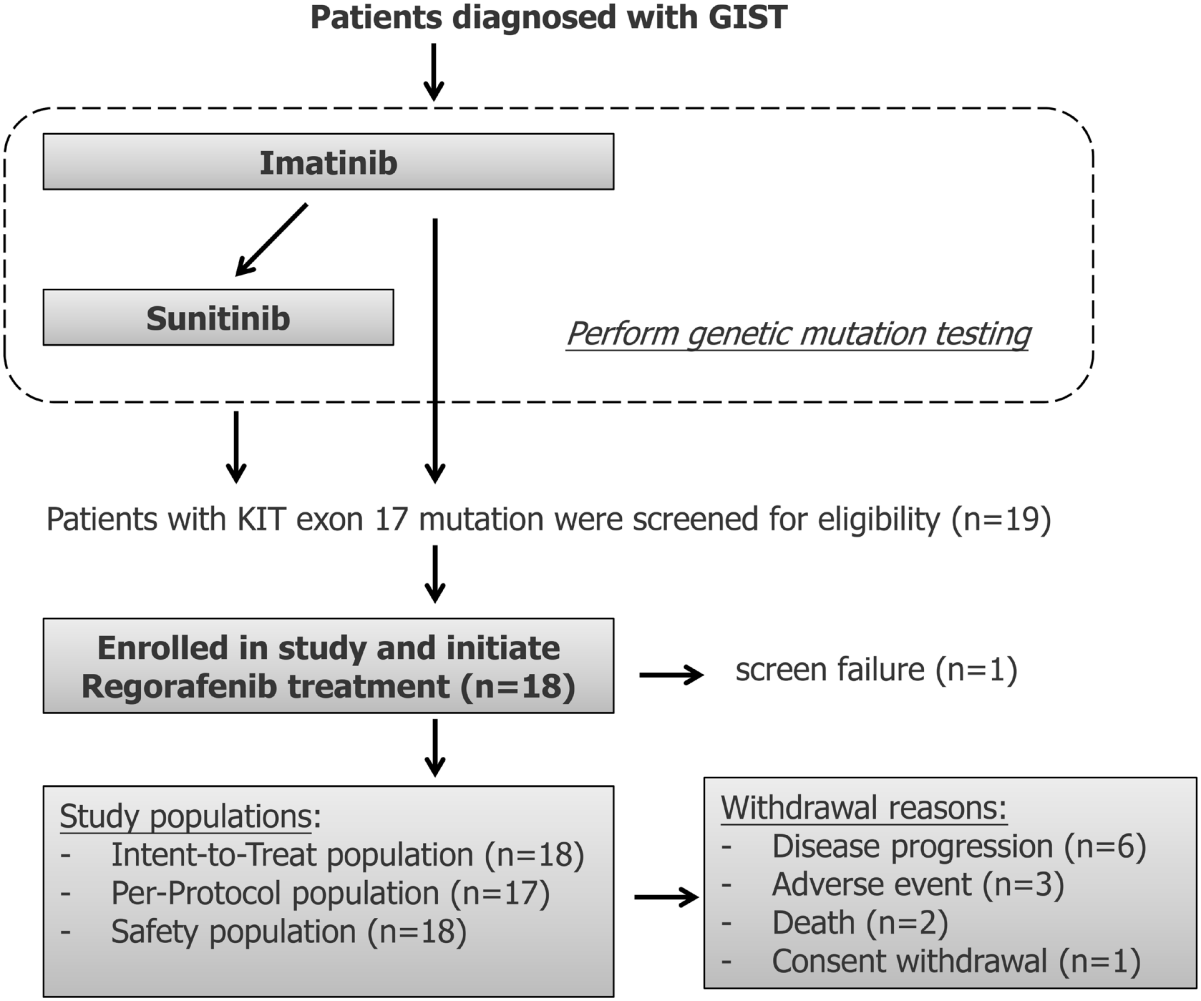
Flowchart for patient selection for regorafenib treatment in patients with metastatic and/or unresectable gastrointestinal stromal tumors harboring secondary mutations of exon 17 Intent-to-treat (ITT) population included all enrolled subjects who took at least one dose of study medication and satisfy the eligible criteria. Subjects included in Per-Protocol (PP) population are those who met the following criteria: 1) at least one dose of study medication; 2) satisfy the eligible criteria; 3) without any protocol violation; and 4) with drug compliance greater than or equal to two cycles. population included subjects who took at least one dose of study medication.

**Figure 3:**
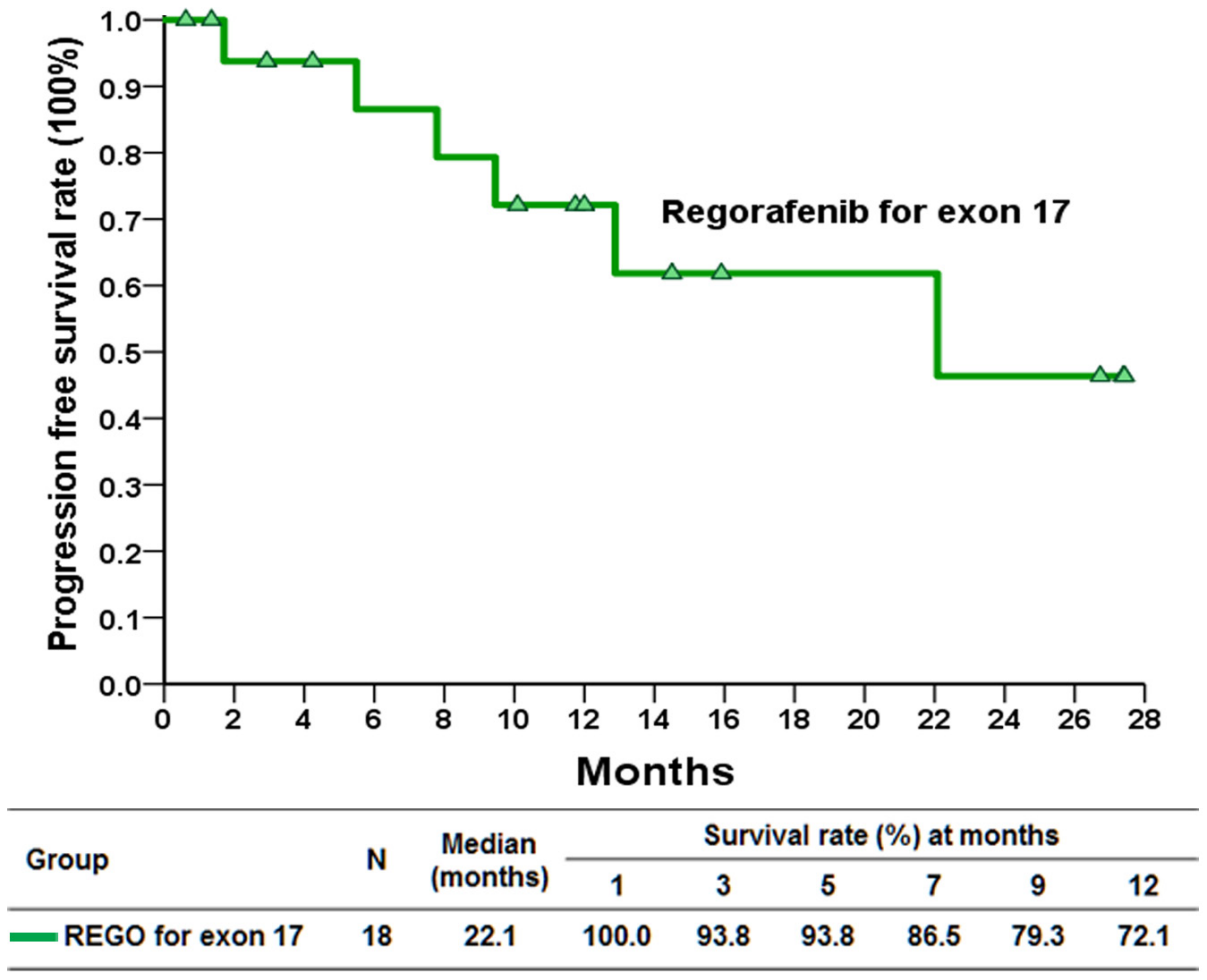
Kaplan–Meier plot of progression free survival in patients with gastrointestinal stromal tumor with exon 17 mutations treated with regorafenib

**Figure 4:**
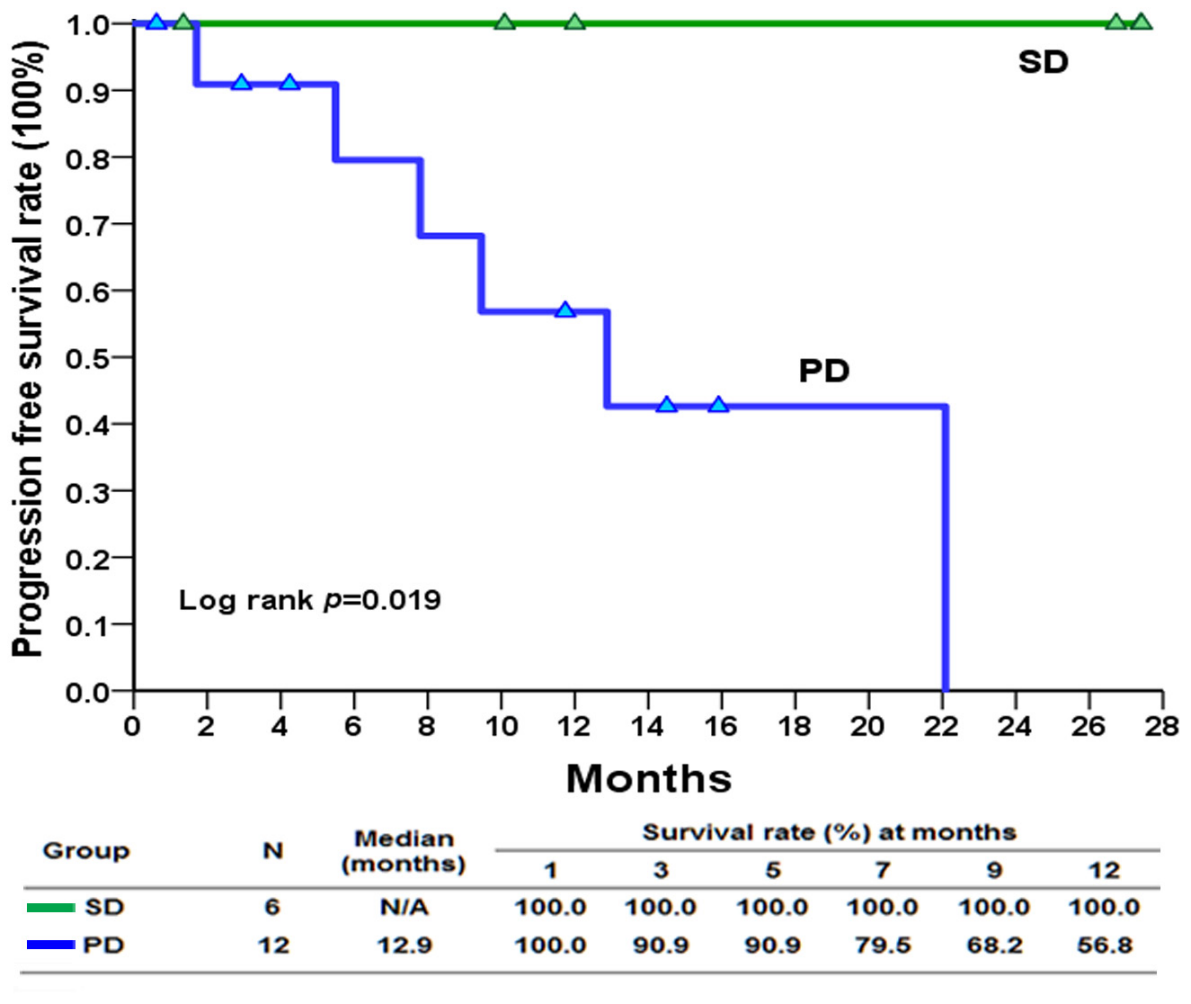
Kaplan–Meier plot of progression free survival in patients with gastrointestinal stromal tumor with exon 17 mutations who had stable disease and progressive disease at enrollment treated with regorafenib

Of the18 patients enrolled, 15 were eligible for response evaluation. Three of the patients were not available for response evaluation, as two were intolerant to regorafenib and one was excluded at the investigator's discretion. The tumor responses of the 15 eligible patients are summarized in Table 1. Fourteen patients (93.3%) achieved a clinical benefit after 16 weeks of treatment. Six patients (40%) had a PR (Figure 1), eight patients (53.3%) exhibited SD, and one patient (6.7%) experienced disease progression. The median treatment duration was 10.0 months (95% confidence interval [CI]: 0.6–24.9 months, Figure 2). In the 18 enrolled patients, the median PFS was 22.1 months (Figure 3). The median overall survival (OS) was not reached during the median follow-up time for 10.9 months (range: 1.0–27.0 months). In the historical cohort group, the PFS after the discovery of exon 17 mutations was 5.5 months (95% CI: 2.85–8.07 months). Therefore, the median PFS was much longer in the patients treated with regorafenib than in the historical patients not receiving regorafenib (22.1 vs. 5.5 months, *p* = 0.0001, Supplementary Figure 1). Moreover, the PFS of the patients who had SD at enrollment was significantly better than that of the patients who had progressive disease (median: not reached vs. 12.9 months, *p* = 0.015, Figure 4).

### Safety

**Figure 5:**
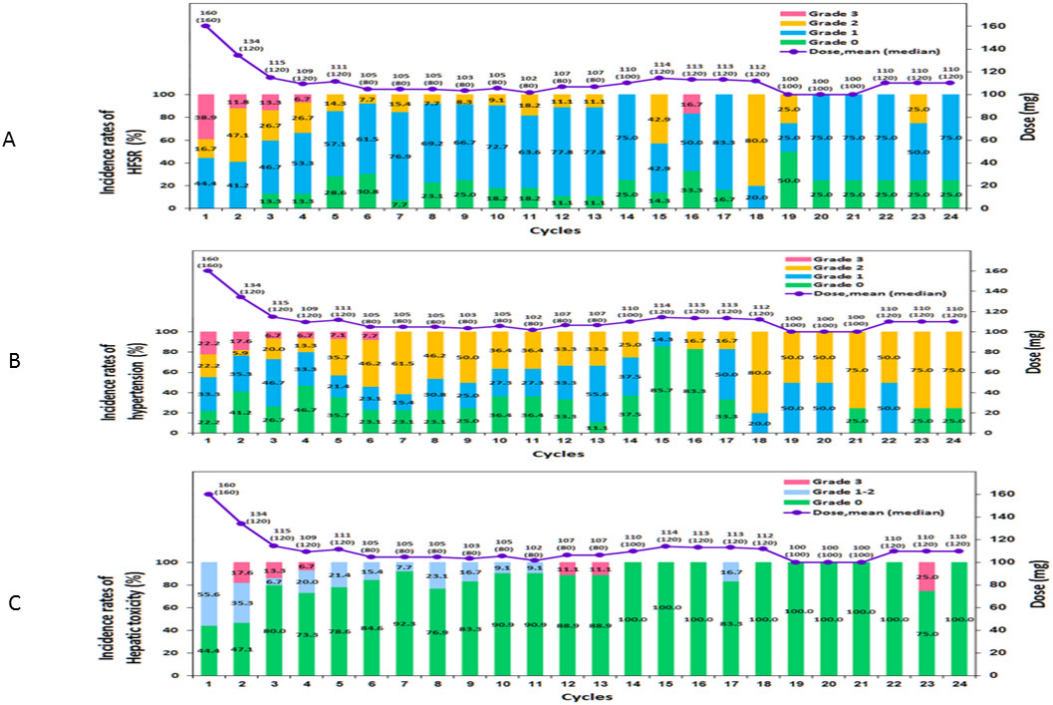
Toxicities of any grade (potentially study drug related) and average/median dose of study, occurring in the 24-month treatment period **(A)** Hand-and-foot skin reactions, **(B)** hypertension, and **(C)** hepatic toxicity.

The mean and median dose of regorafenib per day at 24 weeks was 110 and 120 mg, respectively, as 3 of 18 patients were able to re-escalate the dose (16.7%). Safety was assessed in all 18 patients; the hematological and non-hematological adverse events are listed in Table 2. In this study, the most common grade 3 adverse events were hand-and-foot skin reactions (HFSRs; 55.6%), hypertension (27.8%), hepatic toxicity (16.7%), and fatigue (5.6%) (Figure 5A–5C).

**Table 2:** Adverse events (AEs) from regorafenib treatment for advanced GIST patients harboring exon 17 mutations (*N* = 18)

Side effect		Grade	
	Any Grade	Grade 1-2	Grade 3
Any event	18 (100%)	7 (38.89%)	11 (61.11%)
Hematological AEs			
Anemia	18 (100%)	16 (88.89%)	2 (11.11%)
Leukopenia	0	0	0
Thrombocytopenia	18 (100%)	18 (100%)	0
Non-Hematological AEs			
Hand-foot skin reaction	18 (100%)	8 (44.44%)	10 (55.56%)
Hypertension	16 (88.89%)	11 (61.11%)	5 (27.78%)
Diarrhea	9 (50.00%)	9 (50.00%)	0
Fatigue	10 (55.56%)	9 (50.00%)	1 (5.56%)
Oral mucositis	6 (33.33%)	6 (33.33%)	0
Alopecia	7 (38.89%)	7 (38.89%)	0
Husky voice	6 (33.33%)	6 (33.33%)	0
Anorexia	3 (16.67%)	3 (16.67%)	0
Palpitations	2 (11.11%)	2 (11.11%)	0
Hepatic toxicity	13 (72.22%)	10 (55.56%)	3 (16.67%)
Myalgia	3 (16.67%)	3(16.67%)	0

AEs: adverse events.

## DISCUSSION

The majority of GISTs are caused by the constitutive activation of KIT or PDGFR. IM has been demonstrated to achieve an 80%–90% disease control rate for GISTs due to its selective inhibition of KIT and PDGFR. Eventually, however, resistance to IM typically appears due to secondary mutations in KIT, including mutations in the exons that encode the ATP (and drug) binding pocket (exons 13 and 14) and in the exons encoding the kinase activation loop (exons 17 and 18) [18–19]. Preclinical studies indicate that regorafenib exhibits an inhibitory activity against these activation loop kinase mutations due to its unique structure. Treatment with regorafenib led to decreased phosphorylation of KIT and downstream signaling proteins (including AKT and MAPK) [20]. It is also possible that regorafenib inhibits other signaling pathways (such as the fibroblast growth factor receptor 1 pathway) that may contribute to GIST resistance through previously unrecognized compensatory signaling pathways.

While successful regorafenib treatment of sporadic cases of GIST with exon 17 mutations has been reported in a previous phase II trial [20], the treatment efficacy of regorafenib for patients with GIST with exon 17 mutations is still unknown. A recent follow-up report on the aforementioned phase II trial showed that the median PFS for the sample of only seven patients was 22 months [21]. In this phase II trial, we demonstrated that regorafenib had notable anticancer activity in patients with GIST with exon 17 mutations, particularly when such patients were compared with similar patients treated without regorafenib, with the comparative results indicating that regorafenib significantly prolonged the PFS from 5.5 to 22.1 months (Supplementary Table 1 and Figure 1). The treatment efficacy was also demonstrated by a 93.3% disease control rate after 16 weeks of treatment.

Moreover, while regorafenib is approved as the third-line therapy for imatinib- and sunitinib-resistant GIST patients [17], the subgroup analysis from this phase II trial showed that regorafenib might have a PFS benefit when it is used upfront for patients with GIST harboring exon 17 mutations with SD compared with those experiencing disease progression (median PFS: NR versus 12.9 months, Figure 2). Thus, in patients with GIST with exon 17 mutations, regorafenib might be used irrespective of imatinib or sunitinib use in these patients.

The observed adverse events of treatment in this trial was similar to that reported in previous phase II and III trials of regorafenib, with HFSRs, hypertension, fatigue, and diarrhea being the most common adverse events observed [22–24]. These toxicities are also consistent with the toxicity profile of other kinase inhibitors with a similar target spectrum. Despite the majority of patients requiring at least one dose reduction due toxicity, some patients (3 of 18; 16.67%) were subsequently able to have the regorafenib dose re-escalated without the recurrence of unacceptable adverse effects. More specifically, the mean and median dose at 24 weeks of regorafenib per day was 110 and 120 mg, respectively, since 16.67% patients were able to re-escalate the dose. However, we could not make any conclusions regarding any possible dose–response relationships between regorafenib and adverse events in this study.

HFSRs are not life threatening; however, these adverse reactions are associated with significant tenderness that affect daily functioning and the quality of life, often leading to dose modifications or the discontinuation of treatment [25–27]. As shown in this trial and other previous studies, Asian patients exhibit increased susceptibility to tyrosine kinase inhibitor-induced HFSRs [26–30]. Genetic polymorphisms of TNF-α, VEGF, and UGT1A9 genes have been reported to be a link between HFSRs in patients with hepatocellular carcinoma treated with sorafenib [30]. Moreover, mechanistic studies are urgently needed to shed light on potential solutions for these adverse events.

Although this trial showed that regorafenib significantly prolonged PFS in patients with advanced GIST harboring secondary mutations of exon 17, there are several limitations of this study. First, no large patient cohort with secondary mutations of exon 17 was available for comparison of the treatment efficacy of regorafenib. Thus, a biased historical cohort was chosen for this comparison. Second and the most importantly, the heterogeneity of GIST is a key concern, particularly diagnosis and treatment. Intra-tumor heterogeneity and inter-tumor heterogeneity are always a problem. The heterogeneity of GIST may explain different treatment efficacies of regorafenib between patients harboring secondary mutations of exon 17.

In summary, regorafenib significantly prolonged the PFS in patients with advanced GIST harboring secondary mutations of exon 17. A phase III trial of regorafenib versus placebo is warranted to define the efficacy of regorafenib in this setting.

## MATERIALS AND METHODS

### Study design

This study was an open-label, noncomparative, single-center, and single-arm phase II study evaluating the efficacy of regorafenib for patients with metastatic GIST with KIT exon 17 mutations. The primary endpoint of the study was to determine the overall clinical benefit rate (complete response + PR + SD) for these patients at week 16 after treatment. The secondary endpoints were to determine the objective response rate, the PFS, the OS, and the toxicity levels of the patients. The study was approved by the Institutional Review Board of Chang Gung Memorial Hospital. The study was conducted in full accordance with the International Conference on Harmonization Good Clinical Practice guidelines and the Declaration of Helsinki. All patients provided written informed consent before entering the study. This trial is registered atClinicalTrials.gov, number NCT02606097.

### Eligibility

Patients with a histopathologically proven GIST and a confirmed KIT exon 17 mutation were assessed for eligibility, regardless of whether the disease was progressing or stable. The major inclusion criteria were as follows: at least one measurable disease, age > 20 years, an ECOG PS of 0–1, adequate bone marrow function (defined by a leukocyte count of ≥4000 leukocytes/μl, an absolute neutrophil count of ≥1500 neutrophils/μl, a platelet count of ≥100,000 platelets/μl, and a serum hemoglobin level of ≥9 g/dl), adequate renal function (defined by a serum creatinine level at least 1.5-fold lower than the reference value), and adequate hepatic function (defined by a bilirubin level at least twofold lower than the reference value and aspartate aminotransferase and alanine aminotransferase levels at least 2.5-fold lower than the reference values). Patients with uncontrolled hypertension, bleeding diathesis, or brain metastasis, as well as those who could not take the study medication orally, were excluded.

### Treatment schedule

Eligible patients received 160 mg of oral regorafenib (Stivarga®, Bayer) daily for three consecutive weeks, followed by 1 week without treatment, comprising a 4-week cycle. The treatment was discontinued in the event of disease progression, the occurrence of unacceptable toxic effects, or at the investigator's discretion.

### Response and toxicity evaluation

The response to therapy was assessed by an independent response review committee, based on the results of a radiological evaluation of any measurable lesion every 8 weeks with RECIST version 1.1^15^ using computed tomography or magnetic resonance imaging. After discontinuation of the study treatment, patients were followed up every 3 months until disease progression or death. Toxicity was evaluated and recorded according to version 4.0 of the Common Terminology Criteria for Adverse Events of the National Cancer Institute. All of the patients were included in the toxicity assessment. For the toxicity analysis, the data representing the worst toxicity for each patient from all of the chemotherapy cycles were used.

### Analysis of KIT mutations

Tissue sections were prepared from formalin-fixed, paraffin-embedded, pretreated specimens that were trimmed to enrich tumor cells. Polymerase chain reaction amplification of genomic DNA for KIT was performed, and amplification was analyzed for mutations as previously described [16].

### Statistical analysis

The requirements for a single-stage phase II design were used to determine the number of patients to be enrolled. Assuming a target level of interest of *p*1 = 0.5 (according to a previous phase III GRID trial, the PFS at 4 months was 50%) [17] and a lower activity level of *p* = 0.2, 17 patients would provide an 80% power with a significance level of 0.05. Using these criteria, if clinical benefits are observed in at least 7 of 17 patients, a treatment is considered promising unless other considerations indicate otherwise.

The response and toxicity data were described using simple descriptive statistics. PFS was calculated from the first day of treatment (when the patient was documented with a histopathologically proven GIST and a confirmed exon 17 mutation) until the first day of documented disease progression or death from any cause. PFS was censored at the date of the last follow-up visit for the patients who were still alive and had no documented disease progression. OS was calculated from the first day of treatment until the day of death. PFS and OS were estimated using the Kaplan–Meier method.

### Ethical approval

The study was approved by the Institutional Review Board of Chang Gung Memorial Hospital (IRB number: 103-0111A3. The study was conducted in full accordance with the International Conference on Harmonization Good Clinical Practice guidelines and the Declaration of Helsinki. All patients provided written informed consent before entering the study.

## SUPPLEMENTARY MATERIALS FIGURE AND TABLE


