# Supplementary material for: A phase II trial of regorafenib in patients with metastatic and/or a unresectable gastrointestinal stromal tumor harboring secondary mutations of exon 17
Source: Oncotarget. 2017 Apr 21;8(27):44121–30. doi: 10.18632/oncotarget.17310 (PMC5546467; doi:10.18632/oncotarget.17310)
Supplement: Supplementary file 1 [file oncotarget-08-44121-s001.pdf]

A phase II trial of regorafenib in patients with metastatic and/or a unresectable gastrointestinal stromal tumor harboring secondary mutations of exon 17

SUPPLEMENTARY FIGURE AND TABLE

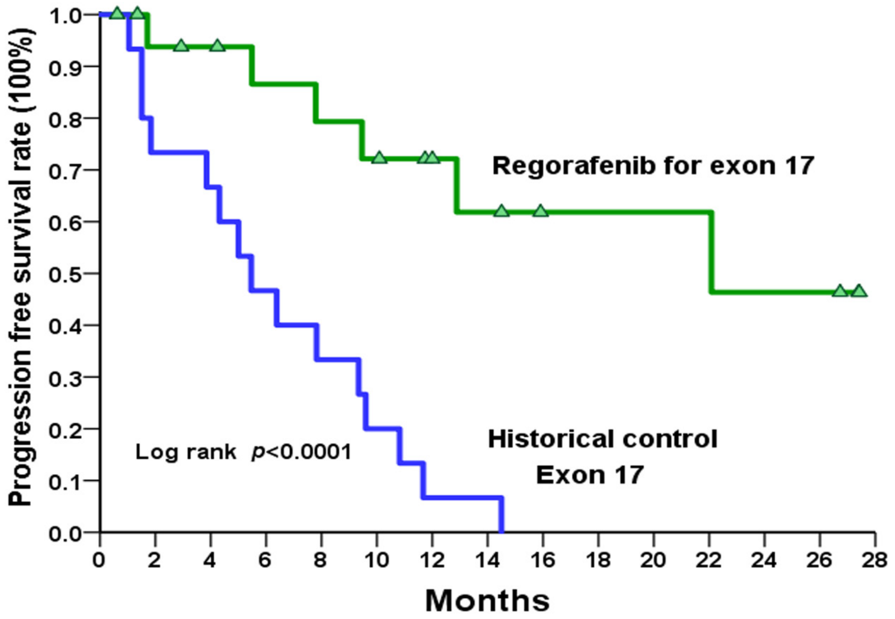

Group	N	Median (months)	Survival rate (%) at months					
			1	3	5	7	9	12
REGO for exon 17	18	22.1	100.0	93.8	93.8	86.5	79.3	72.1
Exon 17	15	5.5	100.0	73.3	53.3	40.0	33.3	6.7

Supplementary Figure 1: Kaplan–Meier plot of progression free survival in patients with gastrointestinal stromal tumor with exon 17 mutations treated with and without regorafenib.

Supplementary Table 1: Demographic data and treatment outcomes for advanced GIST patients harboring exon 17 mutations without regorafenib treatment (*N* = 15)

Median age at time of	
Diagnosis of GIST	59
Diagnosis of metastatic GIST	59
Start of IM	59
Start of SU	62.5
Gender	
Male: Female	10:5
Mutation status	
Exons 11 and 17	10
Exons 9 and 17	2
Exons 11 and 13 and 17	3
No. of operations prior to imatinib therapy	
1/2/3	9/5/1
Adjuvant imatinib use	10
Median duration of imatinib use of advanced disease (m)	48.9
Best response to imatinib	
CR	1
PR	2
SD	10
PD	2
No. of operations on imatinib	
0	3
1	9
2	2
3	1
Management after discovery of exon 17 double mutation	
Maintain imatinib use only	6
Maintain sunitinib use only	3
imatinib with surgery	1
sunitinib with surgery	1
Imatinib or sunitinib with local ablation therapy, such as TACE, RFA	3
Best supportive care	1
Median progression-free survival (m)	5.46

TACE: transcatheter arterial chemoembolization; RFA: radiofrequency ablation.
